# Severe Acute Respiratory Syndrome Coronavirus 2: From Gene Structure to Pathogenic Mechanisms and Potential Therapy

**DOI:** 10.3389/fmicb.2020.01576

**Published:** 2020-07-03

**Authors:** Jun Wu, Xiaohui Yuan, Bing Wang, Rui Gu, Wei Li, Xuemei Xiang, Lijun Tang, Hongyu Sun

**Affiliations:** ^1^Department of Basic Medical Sciences, The General Hospital of Western Theater Command, Chengdu, China; ^2^College of Medicine, Southwest Jiaotong University, Chengdu, China

**Keywords:** SARS-CoV-2, COVID-19, gene structure, protein function, pathogenic mechanisms, potential therapeutic targets

## Abstract

Severe acute respiratory syndrome coronavirus 2 (SARS-CoV-2) is a newly emerging respiratory virus with high morbidity, which was named coronavirus disease 2019 (COVID-19) by World Health Organization (WHO). COVID-19 has triggered a series of threats to global public health. Even worse, new cases of COVID-19 infection are still increasing rapidly. Therefore, it is imperative that various effective vaccines and drugs should be developed to prevent and treat COVID-19 and reduce the serious impact on human beings. For this purpose, detailed information about the pathogenesis of COVID-19 at the cellular and molecular levels is urgently needed. In this review, we summarized the current understanding on gene structure, protein function, and pathogenic mechanisms of SARS-CoV-2. Based on the above, we refined the correlations among gene structure, protein function, and pathogenic mechanisms of SARS-CoV-2. Importantly, we further discussed potential therapeutic targets, aiming to accelerate the advanced design and development of vaccines and therapeutic drugs against COVID-19.

## Introduction

On January 7, 2020, severe acute respiratory syndrome coronavirus 2 (SARS-CoV-2) was identified as the etiological agent of a novel pneumonia that emerged in December 2019, in Wuhan City, Hubei province in China (Lu H. et al., 2020). This novel pneumonia was named coronavirus disease 2019 (COVID-19) by World Health Organization (WHO) (Sohrabi et al., 2020). According to the analysis of genomic structure of SARS-CoV-2, it belongs to β-coronaviruses (CoVs) (Chan et al., 2020; Lu R. et al., 2020). As we know, CoVs belong to the subfamily Coronavirinae, family Coronaviridae, order *Nidovirales*. In this subfamily, there are four CoVs: α-CoV, β-CoV, δ-CoV, and γ-CoV (Chen Y. et al., 2020). To date, there are 7 CoVs that can infect human, including 2 α-CoV (HCoV-229E and-HKU-NL63) and 5 β-CoV (HCoV-OC43, HCoV-HKU1, severe acute respiratory syndrome CoV (SARS-CoV), Middle East respiratory syndrome CoV (MERS-CoV), and SARS-CoV-2) (Chan et al., 2020). Unpredictably, 3 of 7 CoVs cause serious disease with highly contagious among humans, namely SARS-CoV, MERS-CoV, and SARS-CoV-2, resulting in severe disasters and losses of humanity.

On March 11, 2020, WHO declared COVID-19 outbreak as a global pandemic (Cucinotta and Vanelli, 2020). So far, the confirmed cases have exceeded 6,000,000 and the death cases have exceeded 300,000. Even worse, the number of infections is still increasing rapidly every day. Therefore, it is imperative that various effective vaccines and drugs should be developed to prevent and treat COVID-19 and reduce the serious impact on human beings. For this purpose, detailed information about the pathogenesis of COVID-19 at the cellular and molecular levels is urgently needed. In this review, we summarized the current understanding of gene structure, protein function and pathogenic mechanisms of SARS-CoV-2, Based on the above, we refined the correlations among gene structure, protein function, and pathogenic mechanisms of SARS-CoV-2. Importantly, we further discussed potential therapeutic targets, aiming to accelerate the advanced design and development of vaccines and therapeutic drugs against COVID-19.

## Genomic Structure OF SARS-CoV-2

**Figure 1:**
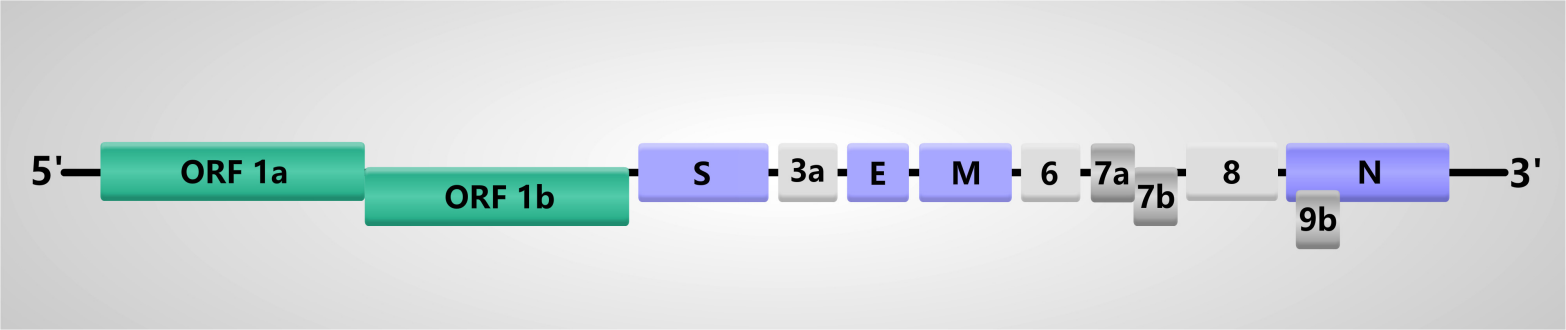
Genome structure of SARS-CoV-2. The SARS-CoV-2 genome comprises of the 5′-untranslated region (5′-UTR), open reading frame (ORF) 1a/b encoding non-structural proteins (nsp), structural proteins including spike (S), envelop(E), membrane(M), and nucleocapsid(N) proteins, accessory proteins such as ORF3a, 6, 7a, 7b, 8 and 9b, and the 3′-untranslated region (3′-UTR).

The genome of SARS-CoV-2 is a single-stranded positive-sense RNA (+ssRNA) with the size of 29.8–30 kb encoding about 9860 amino acids (Chan et al., 2020; Kim et al., 2020). SARS-CoV-2 is a polycistronic mRNA with 5′-cap and 3′-poly-A tail. Their order in the genome is 5′-replicase (open reading frame (ORF)1/ab)-structural proteins [Spike (S)-Envelope (E)-Membrane (M)-Nucleocapsid (N)]-3′ and lacks the hemagglutinin-esterase gene (Figure 1) (Chan et al., 2020; Chen Y. et al., 2020; Kim et al., 2020). This genomic structure is similar to other β-CoVs, so we can infer the translation mechanism of SARS-CoV-2 based on the previous and current experimental evidence. The specific mechanism is listed as follows. Firstly, the genomic RNA is translated into polyprotein 1a/1ab directly, which forms the replication-transcription complex in a double-membrane vesicle. Subsequently, a nested set of subgenomic RNAs are synthesized by a replication-transcription complex in a manner of discontinuous transcription (Hussain et al., 2005; Snijder et al., 2006). Reportedly, there are at least six ORFs in the genome and subgenomes of a typical CoV (Chen Y. et al., 2020). Transcription regulatory sequences located between ORFs are necessary for transcription termination and subsequent acquisition of a leader RNA. The SARS-CoV-2 has 12 functional ORFs and 9 transcription-regulatory sequences. These ORFs can express a total of 16 non-structural proteins (nsp), 4 structural proteins and some accessory proteins, namely, nsp1-16, S, E, M, N, ORF3a, ORF6, ORF7a/b, ORF8, and ORF9b proteins (Chan et al., 2020). Actually, the first ORF, which is about two-thirds of the whole genome length and encodes a set of nsps, expresses two polypeptides: pp1a and pp1ab. Then these two polypeptides are cleaved into 16 nsps by virally encoded chymotrypsin-like protease or main protease and one or two papain-like proteases (Ziebuhr et al., 2000; Masters, 2006). With regard to the structural genes S, E, M, and N, SARS-CoV-2 prefers pyrimidine rich codons to purines. Most high frequency codons were ending with A or T, while the low frequency and rare codons were ending with G or C (Kandeel et al., 2020), indicating that these structural genes have higher gene expression efficiency.

Of note, viral RNA modification is important to regulate the expression of gene, including N6-methyladenosine (m6A), 5-methylcytosine methylation (5 mC), 2-O-methylation (Nm), deamination, and terminal uridylation. In SARS-CoV-2 genome, 41 potential modification sites were found and the most frequently observed motif is AAGAA (Kim et al., 2020). However, the type of modification(s) is yet to be identified. Thus, exploring the SARS-CoV-2 RNA modification should be undertaken, which may reveal the new patterns of gene expression regulation.

In the RNA secondary structures, the SARS-CoV-2 5′-UTR (untranslated regions) contains stem-loops (SL) 1, SL2, SL3, SL4, S5, SL5A, SL5B, and SL5C structures that are similar among the SARS-CoV-2, human SARS-CoV and the bat SARS-related ZC45, and contains SL6, SL7, SL8, and an additional SL which are the same as SARS-CoV. Part of the S5 found was inside the ORF1a/b of the SARS-CoV-2, but the S5 was not found inside the ORF1a/b of SARS-related CoV ZC45. And bat SARS-related CoV ZC45 did not have the SARS-COV SL6-like additional SL. The SARS-CoV-2 had various 3′-UTR structures, including BSL, S1, S2, S3, S4, L1, L2, L3, and HVR. The 3′-UTR was conserved among SARS-CoV-2, human SARS-CoV, and SARS-related CoVs (Yang and Leibowitz, 2015; Chan et al., 2020).

## The Role of SARS-CoV-2 Proteins

To date, the SARS-CoV-2 has been discovered for <4 months, so that the studies about the role of its proteins are lacking according to the knowledge. Here, we reviewed the current knowledge of SARS-CoV-2 proteins, especially the comparison with other CoVs, and highlighted the structural differences of SARS-CoV-2 from other CoVs in order to understand SARS-CoV-2 better (Table 1).

**Table 1:** The structural differences of SARS-CoV-2 proteins relative to other CoVs based on current understanding.

**Protein**	**Structural differences**
S protein	In RBD, compared with SARS-CoV, the asparagine (N439 in SARS-CoV-2) replaces arginine (R426 in SARS-CoV RBD), and a lysine (K417 in SARS-CoV-2) replacement of valine (V404 in SARS-CoV) on β6 formed an extra salt bridge with D30 on ACE2 (Tian et al., 2020). In RBM, compared with SARS-CoV, a one-residue is inserted on a loop away from the ACE2-binding region. In addition, SARS-CoV-2 RBM contains structural changes in the hACE2-binding ridge, largely caused by a four-residue motif (residues 482–485: Gly-Val-Glu-Gly). This structural change allows the ridge to become more compact and form better contact with the N-terminal helix of hACE2 (Wan et al., 2020; Yan et al., 2020)
RdRp	Compared with SARS-CoV, there is a new β-hairpin domain at the N terminus of SARS-CoV-2 (Gao et al., 2020)
Mpro	Compared with SARS-CoV, the threonine (Thr285 in SARS-CoV Mpro) is replaced by alanine (Ala285 in SARS-COV-2 Mpro) and the isoleucine by leucine; and the replacing Ser284, Thr285, and Ile286 by alanine residues may enhance its catalytic activity (Lim et al., 2014; Zhang et al., 2020d)
Nsp2	The amino acid in position 321 has a polar amino acid (glutamine amino acid) (Angeletti et al., 2020)
Nsp3	Compared with Bat SARS like and SARS CoVs, the amino acid in position 543 displays a serine replacing for glycine. Regarding the amino acid in position 192, the homologous region of the Bat SARS-like CoV and SARS-CoV have a polar and an apolar amino acid, respectively, while the SARS-CoV-2 has proline (Angeletti et al., 2020)
ORF8	Compared with SARS-CoV, lacking an aggregation motif VLVVL (amino acid 75–79) in SARS-CoV-2 (Chan et al., 2020)
M protein	Having higher gene expression efficiency compared with SARS, bat SARS and MERS CoV (Kandeel et al., 2020)
N protein	Having higher gene expression efficiency compared with SARS, bat SARS, and MERS CoV (Kandeel et al., 2020)
E protein	Having higher gene expression efficiency compared with SARS, bat SARS, and MERS CoV (Kandeel et al., 2020)

### Structural Proteins

At present, proteins S, E, M, and N are considered as the essential structure proteins for virus assembly and infection of CoVs.

Among them, S protein is critical for SARS-CoV-2 infection. S protein consists of receptor-binding S1 and membrane-fusion S2 subunits, which is responsible for attachment to the host receptor and fusion with cell membrane (Li, 2016; Shang et al., 2020). Its functional domains include N-terminal domain, receptor-binding domain (RBD), and receptor-binding motif (RBM) in S1 subunit and fusion peptide, heptad repeat (HR) 1, HR2, transmembrane domain, and cytoplasm domain in S2 subunit via amino acid sequence alignment (Li, 2016; Lu R. et al., 2020; Wan et al., 2020). And HR1 and HR2 domains are the “fusion core region” of SARS-CoV-2 (Xia et al., 2020b). The receptor of SARS-CoV-2 is the same as SARS-CoV, namely angiotensin-converting enzyme 2 (ACE2), by analyzing S protein domains and the structure of ACE2 (Lu R. et al., 2020; Wan et al., 2020). At present, several studies have analyzed the S protein structure of SARS-CoV-2. The S proteins of SARS-CoV-2 and SARS-CoV have an amino-acid sequence identity of around 77% (Zhou et al., 2020), indicating the existence of cross-reaction. Two studies reported that SARS-CoV-specific neutralizing antibody, CR3022, could bind to SARS-CoV-2 RBD, confirming the existence of cross-reaction (Tian et al., 2020; Yuan et al., 2020). However, other SARS-CoV-specific neutralizing antibodies (e.g., m396, CR3014) that target the ACE2 binding site of SARS-CoV failed to bind with SARS-CoV-2 S protein, implying that the difference in the RBD of SARS-CoV and SARS-CoV-2 (Tian et al., 2020). Further analysis on RBD of both two viruses showed the arginine (R426 in SARS-CoV RBD) to asparagine (N439) mutation in SARS-CoV-2, abolishing the strong polar interactions; and a replacement from valine (V404 in SARS-CoV) to lysine (K417 in SARS-CoV-2) on β6 formed an extra salt bridge with D30 on ACE2 (Tian et al., 2020). Interestingly, CR3022 can bind to RBD of SARS-CoV-2 due to the existence of a highly conserved cryptic epitope in RBD of SARS-CoV-2 and SARS-CoV (Yuan et al., 2020). Moreover, only when the RBD is in the “up” conformation, the CR3022 can bind to RBD. CR3022 Fab binds to SARS-CoV RBD with a much higher affinity than to SARS-CoV-2 RBD. The difference in binding affinity of CR3022 between SARS-CoV-2 RBD and SARS-CoV RBD may be due to the non-conserved residues in the epitope (Yuan et al., 2020). Chan et al. found that the S2 subunit of SARS-CoV-2 was highly conserved and shared 99% identity with those of the two bat SARS-like CoVs (SL-CoV ZXC21 and ZC45) and human SARS-CoV (Chan et al., 2020); and bat SARS-like CoVs have two deletion of RBD in S protein. These studies suggest that SARS-CoV-2 can cross the species barriers, making it easier to spread among human beings.

The structural changes of RBM can make the SARS-CoV-2 more favorable for binding with ACE2. Compared with SARS-CoV, SARS-CoV-2 RBM contains structural changes in the hACE2-binding ridge, which are largely caused by a four-residue motif (residues 482–485: Gly-Val-Glu-Gly). This structural change allows the ridge to become more compact and form better contact with the N-terminal helix of hACE2 (Yan et al., 2020). Besides, the RBM of SARS-CoV-2 has a one-residue insertion on a loop away from the ACE2-binding region (Wan et al., 2020).

The E protein functions in virus assembly and comprises ion channel actions to help release (Ruch and Machamer, 2012). The M protein can promote membrane curvature and bind to the nucleocapsid (Neuman et al., 2011). And the N protein contains two structurally independent RNA binding domains, the N-terminal RNA binding domain and a C-terminal domain, which can interact with the viral RNA to form the ribonucleoprotein (Risco et al., 1996). Moreover, N protein is a repressor of RNA interference (Cui et al., 2015) and an antagonist of interferon (Lu et al., 2011). Compared with SARS, bat SARS and MERS CoV, protein S, E, M, and N of SARS-CoV-2 have higher gene expression efficiency (Kandeel et al., 2020). However, the structure and role of protein E, M, N need to be further investigated in the future, in order to understand the biological behaviors better.

### Non-structural Proteins

Reportedly, nsp1-16 mainly function in replication (Egloff et al., 2004; Graham et al., 2005; Gadlage et al., 2008; Huang et al., 2011; Angelini et al., 2013; Zeng et al., 2018; Jia et al., 2019), polypeptides cleaving (Zhu et al., 2017; Lei et al., 2018) and inhibiting host immune response (Gadlage et al., 2008; Huang et al., 2011; Zhu et al., 2017; Lei et al., 2018; Shi et al., 2019) of CoVs. As a member of the coronavirus family, the structure of the SARS-CoV-2 nsps is generally similar to other CoVs, but there are some new features.

Angeletti et al. displayed the I-Tasser model of the SARS-CoV-2 nsp2 and nsp3 (Angeletti et al., 2020). Compared with the Bat SARS-like coronavirus, the amino acid of nsp2 in position 321 is a polar amino acid (glutamine amino acid), so nsp2 of SARS-CoV-2 may have higher stability due to its side chain length, polarity, and potential to form H-bonds. The amino acid of nsp3 in position 543 displayed a serine replacing for glycine compared with Bat SARS like and SARS coronaviruses. Regarding the amino acid of nsp3 in position 192, the homologous regions of the Bat SARS-like coronavirus and SARS-CoV have a polar and an apolar amino acid, respectively, while the SARS-CoV-2 has proline. This mutation is located near the protein similar to a phosphatase present also in the SARS coronavirus (PDB code 2acf) playing a key-role in the replication process of the virus in infected cells. This study demonstrates that the structure of nsp2 and nsp3 enables SARS-CoV-2 with enhanced stability and infectivity.

RNA-dependent RNA polymerase (RdRp), namely nsp12, plays a critical role in replication, and transcription of SARS-CoV-2 (Gao et al., 2020). Nsp7 and nsp8 form nsp12-nsp7-nsp8 complex as the co-factors. The structure of the SARS-CoV-2 nsp12 contains a “right hand” RdRp domain (residues S367-F920) and a nidovirus-unique N-terminal extension domain (residues D60-R249) that adopts a nidovirus RdRp-associated nucleo-tidyltransferase (NiRAN) architecture. The architecture of the polymerase core of the viral polymerase family is conserved but there is a newly identified β-hairpin domain at its N terminus in RdRp (Gao et al., 2020).

Main protease (Mpro, 3CLpro), namely nsp5, is essential for processing the polyproteins that are translated from the viral RNA (Zhu et al., 2017). The analysis of crystal structure found that it had the 96% sequence identity compared with SAR-CoV Mpro (Zhang et al., 2020b). In SARS-CoV-2, the threonine is replaced by alanine and the isoleucine by leucine. Importantly, replacing Ser284, Thr285, and Ile286 by alanine residues in SARS-CoV Mpro can lead to a 3.6-fold enhancement of the catalytic activity of the protease (Lim et al., 2014), indicating that SARS-CoV-2 is more active than SARS-CoV.

Currently, the reports about the specific structure and role of nsps are few. The further investigations should focus on replication, polypeptides cleaving and inhibiting host immune response to understand SARS-CoV-2 fully and help seek potential therapeutic targets.

### The Putative Proteins of SARS-CoV-2

ORF3b was found a new putative short protein by Chan and his colleagues in SARS-CoV-2. They found this new protein has 4 helices and no homology in SARS-CoV or SARS-related-CoV (Chan et al., 2020). The function of this protein remains unknown, but we have to attach importance to its role because it may play a significant role in viral pathogenicity based on the understanding of ORF3b in SARS-CoV. Khan et al. transfected ORF3b to Vero E6 cells, and found that necrosis and apoptosis began to occur in these cells after 6 h (Khan et al., 2006). Meanwhile, ORF3b is also an IFN antagonist though inhibiting its synthesis (Kopecky-Bromberg et al., 2007). However, by using two complementary sequencing approaches, direct RNA sequencing (DRS), and sequencing-by-synthesis (SBS), Kim et al. did not find ORF3b mRNA in SARS-CoV-2 (Kim et al., 2020). Therefore, this putative novel short protein needs more evidence to prove its existence.

ORF8 is found in β-coronavirus lineage B coronaviruses, which acts as an accessory protein. In patients with early-phase SARS, the full-length ORF8 can be isolated completely, while it has a 29-nucleotide deletion in mid- and late- phase patients, resulting in producing ORF8a and ORF8b. In ORF8b of SARS-CoV, there is an aggregation motif VLVVL (amino acid 75–79), which can trigger intracellular stress pathways and activate NLRP3 inflammasomes (Kopecky-Bromberg et al., 2007). However, this motif is absent in SARS-CoV-2, so it is presumed to be a “novel” protein of SARS-CoV-2 (Chan et al., 2020). Chan et al. made a prediction about its secondary structure, and found that this putative “novel” protein had a high possibility to form a protein with an alpha-helix, following with a β-sheet(s) containing six strands (Chan et al., 2020). Therefore, ORF8 of SARS-COV-2 is a noteworthy protein in pathogenesis and drug development. However, its function needs further investigations.

Interestingly, Kim et al. found an ORF10 read by DNA nanoball sequencing (DNB-seq) based on the SBS principle. However, this read was not supported by DRS data and ORF10 did not show significant homology to known proteins (Kim et al., 2020). Thus, it should be ascertained whether SARS-CoV-2 expresses ORF10. At least, the annotation of ORF10 should be clear in order to understand SARS-CoV-2 fully.

## The Pathogenic Mechanisms of SARS-CoV-2

### Cellular Entry of SARS-CoV-2

An important process, cellular entry of SARS-CoV-2, is its membrane fusion with the target cell and this process is structural rearrangement of S protein actually (Walls et al., 2020; Wrapp et al., 2020; Xia et al., 2020b; Yan et al., 2020; Zhang et al., 2020d). Firstly, RBD of S1 subunit binds to the peptidase domain (PD) of ACE2, resulting in the three-RBD up conformation, and subsequent shedding of S1 and refolding of S2 subunit. Then, the three HR1 regions assemble into a coiled-coil trimer and three HR2 regions bind to the hydrophobic grooves of the HR1 trimer in an antiparallel manner to form six-helical bundle (6-HB). Finally, this structural rearrangement brings the viral and cellular membranes in close proximity for fusion. After that, the Mpro is essential for processing the polyproteins that are translated from the viral RNA. Recently, two studies about the cryo-EM structure of homotrimeric SARS-CoV-2 S protein demonstrated that the RBD could undergo a hinge-like movement to transition between “up” or “down” conformations (Walls et al., 2020; Wrapp et al., 2020). Only when the RBD is in “up” conformation, the receptor ACE2 can interact with RBD. Moreover, ACE2 bound to the SARS-CoV-2 S protein ectodomain with ~15 nM affinity, indicating that the receptor-binding ability of SARS-CoV-2 is 10–20 times stronger than that of SARS-CoV (Wrapp et al., 2020). Wang et al. found that the key residue substitutions in SARS-CoV-2-CTD slightly strengthened the interaction and led to higher affinity for receptor binding (Wang Q. et al., 2020). Yan et al. found that two S protein trimmers could bind to an ACE2 dimer simultaneously by the structural analysis of full-length human ACE2 (Yan et al., 2020). In addition, the serine protease TMPRSS2 contributes to priming of the SARS-CoV-2 S protein (Hoffmann et al., 2020). Currently, it has been determined that lung type II pneumocytes express ACE2 and TMPRSS2 simultaneously (Ziegler et al., 2020), and TMPRSS2-expressing cells are highly susceptible to SARS-CoV-2 infection (Matsuyama et al., 2020). Ou et al. found that the entry of SARS-CoV-2 S protein into 293/hACE2 cells was mainly mediated through endocytosis (Ou et al., 2020). However, the role of other structural proteins in cellular entry of SARS-CoV-2 is unclear and the role of lipids and calcium in membrane fusion also needs to be studied.

### SARS-CoV-2 Induced Immune Response

After entering the body, SARS-CoV-2 can activate innate and adaptive immune responses and the activation of immune responses may result in lymphocytopenia, exhausted cytotoxic lymphocytes, and cytokine storm. In non-severe COVID-19 patients, Thevarajan et al. detected immunoglobulin M (IgM) and IgG antibodies that bound to SARS-CoV-2 in blood, and found that CD4+ T cells and CD8+ T cells were activated (Thevarajan et al., 2020). In severe COVID-19 patients [Diagnostic criteria: meeting one of three criteria: (1) dyspnea, RR>30 times/min, (2) oxygen saturation <93% in ambient air, (3) PaO2/FiO2<300 mmHg (Wang Y. et al., 2020)], the number of CD4+ T cells, CD8+ T cells, B cells and natural killer (NK) cells, as well as the percentage of monocytes, eosinophils and basophils were reduced significantly (Huang et al., 2020; Qin et al., 2020; Xu et al., 2020). A retrospective study found that 80% of critically ill patients [Diagnostic criteria: meeting one of three criteria: (1) respiratory failure, (2) septic shock, (3) multiple organ failure (Wang Y. et al., 2020)] had lymphopenia, while only 35% of non-critically ill patients had lymphopenia (Yang et al., 2020). Moreover, the neutrophil count and neutrophil- to- lymphocyte ratio were increased in COVID-19 patients, indicating higher disease severity and poor clinical outcome (Zhang et al., 2020b). Besides, the exhaustion markers, NKG2A, on NK cells and CD8+ T cells, were upregulated in COVID-19 patients (Zheng et al., 2020), indicating the exhausted cytotoxic lymphocytes. High-dimensional immune profiling by mass cytometry found that, compared with healthy donors, the proportions of B cells, CD4+CD8+ double-positive T cells (DPTs), naïve CD4+ T cells, and TGF-β+CD28- naïve CD4+ T cells in infected patients were generally increased, whereas CD8+ T cells, regardless of whether they belonged to the effector, naïve, or memory subsets, declined constantly during the progression of infection (Wang W. et al., 2020). Additionally, the proportions of dendritic cells (DCs), macrophages, CD4+ T cells, and TGF-β+CD28- naïve CD8+ T cells were higher in the mild group than in the severe group (Wang W. et al., 2020). Of note, the proteomic and metabolomic data of COVID-19 patient sera showed the dysregulation of some lipids and apolipoproteins associated with macrophage functions, such as sphingolipids, phosphocholine, glycerophospholipids and AOPA1, suggesting the dysregulation of macrophage in COVID-19 patients (Shen B. et al., 2020). These evidences indicated the immunosuppression and dysfunction of immunity as the disease progresses in COVID-19 patients.

There are higher expression levels of proinflammatory cytokines including IL-2, IL-7, IL-6, G-CSF, IP-10, MCP-1, MIP-1A, and TNFα in severe COVID-19 patients, indicating that the cytokine storm was caused (Huang et al., 2020; Ruan et al., 2020). As we all know, so-called cytokine storm can lead to viral sepsis, inflammatory-induced lung injury, pneumonitis, acute respiratory distress syndrome (ARDS), respiratory failure, shock, organ failure, and potential death and also mediate massive infiltration of neutrophils and macrophages, diffuse alveolar damage with the formation of hyaline membranes and a diffuse thickening of the alveolar wall (Huang et al., 2020; Xu et al., 2020). However, the secretion of T-helper-2 (Th2) cytokines, such as IL-4 and IL-10, was increased, which could suppress inflammation. Therefore, the role of Th1 and Th2 responses warrants further investigations (Huang et al., 2020). Reportedly, Th17 cells were increased significantly in peripheral blood cells of severe COVID-19 patients (Wu and Yang, 2020), which may be one of reasons inducing “cytokine storm.” In addition, the cytokine levels are positively correlated with disease severity (Chen L. et al., 2020; Huang et al., 2020). The serum levels of IL-2R, IL-6, IL-7, G-CSF, IP-10, MCP-1, MIP-1A, and TNF-α in severe patients are higher than those in non-severe patients.

Interestingly, Kanduc et al. found that there were vast peptides sharing between SARS-CoV-2 S glycoprotein and surfactant-related proteins (Kanduc and Shoenfeld, 2020), indicating that these shared peptides may trigger cross-reactions. This may be one reason why SARS-CoV-2 prefers to attack the respiratory system.

### SARS-CoV-2 Induced Multiple Organ Function Damage

**Figure 2:**
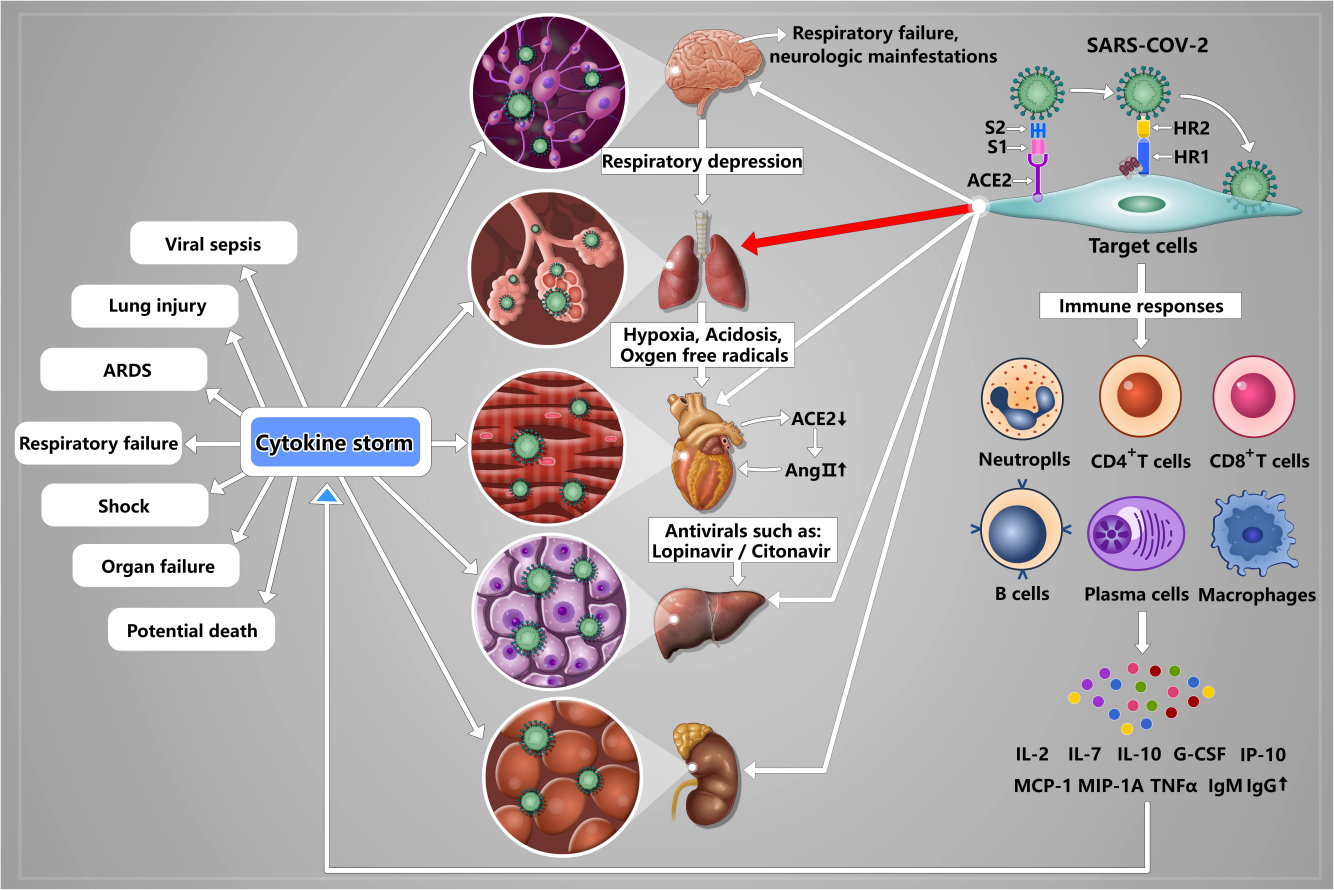
A schematic model of SARS-CoV-2 pathogenesis. Once SARS-CoV-2 enters into the lung by airway, S1 subunit of S protein can bind to the receptor ACE2 expressing on II alveolar epithelial cells, and induce conformational change of the S2 subunit, triggering the association between the heptad repeat (HR) 1 and HR2 domains to form 6-HB, thus bring the viral and cellular membranes in close proximity for fusion, resulting in lung damage that is the main infection site. Upon lung infection, a series of immune responses are induced, including activation of CD4+ and CD8+ T cells, lymphopenia, exhausted cytotoxic lymphocytes, increased IgM and IgG, and strong proinflammatory cytokine storm (IL-2, IL-7, IL-10, G-CSF, IP-10, MCP-1, MIP-1A, and TNF-α), ultimately resulting in viral sepsis, inflammatory-induced lung injury, pneumonitis, acute respiratory distress syndrome (ARDS), respiratory failure, shock, organ failure, and potential death. Meanwhile, SARS-CoV-2 also can directly affect other organs including brain, liver, kidney, and heart via binding to the potential receptor ACE2 expressing on glial cells and neurons, liver cells and bile duct cells, renal tubular cells and myocardial cells. Specifically, (I) In brain, SARS-CoV-2 binding to glial cells and neurons can induce cerebral damage and neurologic manifestations; (II) In liver, SARS-CoV-2 binding to liver cells and bile duct cells can induce liver dysfunction. And antivirals, such as lopinavir/litonavir, can also lead to livery injury; (III) Kidney may be the target organ of SARS-CoV-2 although the mechanism of kidney injury has not been reported; (IV) In heart, the reduced ACE2 can result in increased AngII indirectly. And AngII plays an important role in promoting the development of cardiovascular disease. And acidosis and the generation of oxygen free radicals caused by hypoxia and hypoxia-reperfusion can aggravate myocardial injury.

Based on above analysis, the uncontrolled inflammatory innate responses and impaired adaptive immune responses in severe COVID-19 patients are ubiquitous and these abnormal immune responses can lead to local and systematic tissue damage. These are consistent with clinical outcomes. According to the retrospective studies and pathological findings, many patients with COVID-19 experienced multiple organ function damage, including acute kidney injury, cardiac injury, liver dysfunction, and cerebral damage (Baig et al., 2020; Xu et al., 2020; Yang et al., 2020). In addition to the abnormal activation of immune response, SARS-CoV-2 also can directly affect these organs including brain, liver, kidney, and heart via binding to the potential receptor ACE2 (Figure 2). Therefore, we specifically describe the current understanding on the pathogenic mechanisms of SARS-CoV-2 behind multiple organ infection as follows. Of note, although ACE2 can be expressed on many type cells, such as II alveolar epithelial cells, glial cells and neurons, myocardial cells, liver cells and bile duct cells, and renal tubular cells, no direct evidence shows that the expression level of ACE2 is associated with the invading ability of SARS-CoV-2.

#### Lung

No doubt, lung is the main target organ of SARS-CoV-2 infection (Yang et al., 2020). In lung, type I and II alveolar epithelial cells can express ACE2. Once SARS-CoV enters into the lung by airway and binds to alveolar cells, the number of ACE2 would reduce, leading to dysfunction of the renin-angiotensin system (RAS), strong inflammation response, and vascular permeability (Imai et al., 2005). Besides, increased MCP-1 can also promote the synthesis of angiotensin II, further aggravating the inflammation (Company et al., 2011; Yang et al., 2020). These processes ultimately can induce pulmonary edema, impair lung function, and even ARDS. These previous studies suggested that SARS-CoV-2 might have similar mechanisms in lung injury. However, these analyses are based on the evidence of SARS-CoV and the function of RAS. Thus, further studies are needed to detect the number of ACE2 after infecting SARS-CoV-2 and to explore the precise mechanism how SARS-CoV-2 interacts with host cells.

#### Brain

Of note, Mao et al. found that about 88% patients among the severe cases displayed neurologic manifestations, such as acute cerebrovascular diseases and impaired consciousness (Mao et al., 2020). On this basis, Li et al. suggested that SARS-CoV-2 may be able to invade nervous system, resulting in respiratory failure, and neurologic manifestations though a systematic review that analyzes the neuroinvasive potential of SARS-CoV-2 based on the evidence of other CoVs (Li et al., 2020). Regarding the specific mechanism responsible for cerebral damage, Li et al. found that the neuroinvasive propensity is a common feature of CoVs. Thus, some researchers attempt to isolate SARS-CoV-2 from the endothelium of cerebral microcirculation, cerebrospinal fluid, glial cells, and neuronal tissue by the autopsies of the COVID-19 patients (Baig et al., 2020). The expression level of ACE2 in central nervous system (CNS) is very low and the route of CoVs entering brain is unknown so far. By analyzing known evidence, Li et al. suggested that CoVs might enter peripheral nerve terminals firstly, and then gain access to the CNS via a synapse-connected route (Li et al., 2020). However, interestingly, the latest study found that ACE2 was expressed in human brain, such as over glial cells and neurons (Baig et al., 2020), indicating that SARS-CoV-2 has neurotropic potential. Baig et al. suggested that SARS-CoV-2 entered brain via circulation and/or an upper nasal trancribrial route (Baig et al., 2020). Given that the high similarity between SARS-CoV and SARS-CoV-2 and the latest evidence, we can confirm that SARS-CoV-2 can enter brain, leading to cerebral damage. Thus, we should attach importance to the impact of SARS-CoV-2 on nervous system in subsequent studies and explore the specific mechanisms behind nervous damage.

#### Liver

Currently, we cannot determine whether the liver dysfunction is caused by SARS-CoV-2 or antiviral drugs. On the one hand, liver cells, and bile duct cells express ACE2 and the ACE2 expression of bile duct cells is higher than that of liver cells (Chai et al., 2020). As we know, bile duct epithelial cells play important roles in liver regeneration and immune response (Banales et al., 2019). These results indicated that SARS-CoV-2 may damage liver function. On the other hand, antivirals, such as lopinavir/litonavir, can lead to livery injury (Fan Z. et al., 2020). Postmortem biopsies from a COVID-19 patient showed moderate microvascular steatosis and mild lobular and portal activity, indicating that the injury could be caused by either SARS-CoV-2 infection or antiviral drugs (Xu et al., 2020). Thus, the underlying mechanisms of liver dysfunction need to be further studied.

#### Kidney

Reportedly, ACE2 is highly expressed in kidneys, especially renal tubular cells (Fan C. et al., 2020), suggesting that kidney may be the target organ of SARS-CoV-2. According to the data from 1,099 COVID-19 patients, the occurrence of acute kidney injury was 0.5%, and the severity rate was 83.3% (Guan et al., 2020). Moreover, SARS-CoV-2 could be detected in the urine samples from some COVID-19 patients (Guan et al., 2020). However, the evidence that SARS-CoV-2 can cause kidney injury directly has not been reported.

#### Heart

In SARS patients, the SARS-CoV can be detected in cardiomyocytes (Oudit et al., 2009). After SARS-CoV infecting the lungs of mice, the expression of ACE2 in myocardial tissues decreased in mRNA and protein levels. The reduced ACE2 can result in increased AngII indirectly. And AngII plays an important role in promoting the development of cardiovascular disease (Oudit et al., 2009). Sodhi et al. found that ACE2 can degrade Des-Arg9-bradykinin. When ACE2 was reduced, the Des-Arg9-bradykinin/BK1 receptor pathway would be over-activated, thereby promoting the occurrence of inflammatory reactions (Sodhi et al., 2018). SARS-CoV-2 may have the similar mechanisms of injury in cardiovascular system, but these need further confirmation. In addition, SARS-CoV-2-induced lung damage can lead to impaired gas exchange and subsequent hypoxemia. Acidosis and the generation of oxygen free radicals caused by hypoxia and hypoxia-reperfusion can aggravate myocardial injury, while hypoxia can also induce inflammatory responses, leading to further aggravation of cardiac tissue damage.

## The Correlations Among Gene Structure, Protein Function, and Pathogenic Mechanisms OF SARS-CoV-2

According to the analysis about gene structure, protein function, and pathogenic mechanisms of SARS-CoV-2 above, we conclude that the correlations among them have following features compared with other CoVs. First, SARS-CoV-2 structural proteins have lower effective number of codons (ENc) values compared with SARS, bat SARS, and MERS CoVs (Kandeel et al., 2020), and lower ENc value indicates a generally higher level of expression (Zhang et al., 2018), suggesting that the structural genes S, E, M, and N have higher expression efficiency. Second, compared with SAR-CoV Mpro, the threonine is replaced by alanine and the isoleucine by leucine in SARS-CoV-2, suggesting that the catalytic activity of the Mpro is more active than SARS-CoV (Zhang et al., 2020c,d). Besides, surface plasmon resonance sensorgram showed that the receptor-binding ability of SARS-CoV-2 is 10–20 times stronger than that of SARS-CoV (Wrapp et al., 2020). As we all know, protein sequences are encoded by genes and the changes in protein sequences may lead to the changes in protein function. We listed the structural differences of SARS-CoV-2 proteins relative to other CoVs based on current understanding in Table 1. However, the relationships between these altered protein sequences and gene sequences remains unclear. This is a very complicated problem that must be solved because changes in protein function can affect the stability, infectivity, and pathogenicity of the virus to a certain extent.

In addition, there are vast peptides shared between SARS-CoV-2 S glycoprotein and surfactant-related proteins (Kanduc and Shoenfeld, 2020). This may be one reason why SARS-CoV-2 prefers to attack the respiratory system. Cell–cell fusion assay showed that SARS-CoV-2 had a superior plasma membrane fusion capacity than SARS-CoV (Xia et al., 2020a). Moreover, a study indicates that SARS-CoV-2 spreading also depends on TMPRSS2 activity (Hoffmann et al., 2020); and TMPRSS2-expressing cells are highly susceptible to SARS-CoV-2 infection (Matsuyama et al., 2020). However, the increased activity of TMPRSS2 after viral infection in patients remains to be studied. The TMPRSS2 enzyme cleavage site sequence of SARS-CoV-2 helps enhance its ability to enter cells, and it is important for spreading among humans and animals. In RaTG13, the coronavirus most closely related to SARS-CoV-2, lacks the multibasic cleavage site. Of note, it has been reported that SARS-CoV-2 could exploit species-specific interferon-driven upregulation of ACE2 to enhance infection *in vitro* (Ziegler et al., 2020). Thus, it is essential to explore how SARS-CoV-2 uses the host immune response to escape immune attacks. These evidences suggest the complexity of the pathogenic mechanism of SARS-CoV-2.

In general, based on above analysis, we can know that SARS-CoV-2 is easier to spread across species and has stronger ability to spread from person to person compared with other CoVs. Moreover, the correlations among gene structure, protein function, and pathogenic mechanisms are complicated, so the specific correlations among them remain unclear and need large number of studies to explore.

## Potential Therapeutic Targets

**Figure 3:**
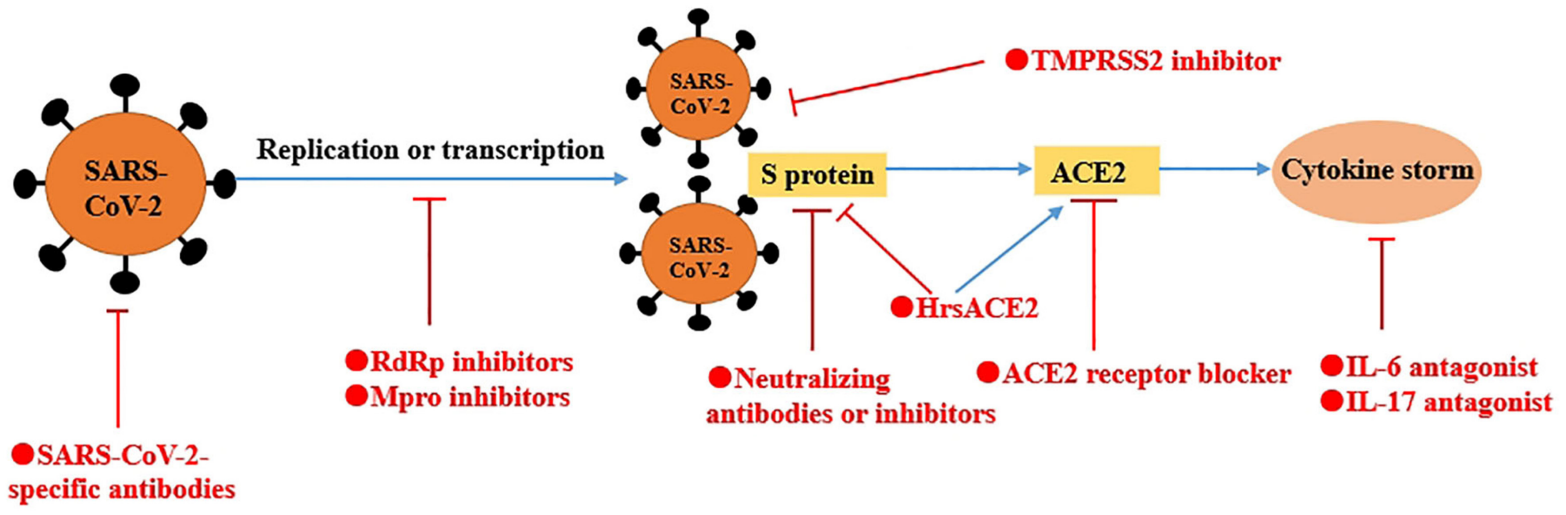
A schematic model of potential therapeutics against COVID-19. Based on the gene structure, protein function, and pathogenic mechanisms of SARS-CoV-2, we proposed some potential therapeutic targets from four aspects, including inhibiting important proteases (e.g., RdRp, Mpro), blocking SARS-CoV-2 from to target cells (e.g., neutralizing antibodies or inhibitors of S protein, ACE2 receptor blocker and TMPRSS2 inhibitor), important targets against “cytokine storm” (e.g., IL-6 and IL-17) and SARS-CoV-2-specific antibodies. In addition, hrsACE2 not only neutralize the virus but also rescue cellular ACE2 activity.

Based on the gene structure, protein function, and pathogenic mechanisms of SARS-CoV-2, we proposed some important potential therapeutic targets from following four aspects, including inhibiting important proteases, blocking SARS-CoV-2 from target cells, important targets against “cytokine storm” and SARS-CoV-2-specific antibodies (Figure 3).

### Inhibiting Important Proteases

#### RdRp

Given the importance of RdRp in replication and transcription of SARS-CoV-2, RdRp looks like an excellent target for new therapeutics. Reportedly, nucleotide analogs, such as remdesivir and sofosbuvir, could inhibit the proliferation of SARS-CoV-2 by binding with its RdRp (Elfiky, 2020a; Wang M. et al., 2020). To this end, Rao et al. further explored the possible binding and inhibition mechanism (Gao et al., 2020). They found that the nsp12 of SARS-CoV-2 had the highest similarity with the Apo state of ns5b. Meanwhile, other antiviral drugs against RdRp also showed the effectiveness, such as galidesivir, tenofovir, and IDX-184 (Elfiky, 2020b; Wang M. et al., 2020). Based on these evidences, exploring the specific inhibitors against SARS-CoV-2 RdRp is essential.

#### Mpro

Due to non-human proteases with a similar cleavage specificity currently, inhibitors of Mpro are unlikely to be toxic. Therefore, Zhang et al. designed an improved α-ketoamide inhibitors to inhibit viral replication (Zhang et al., 2020d). Peptidomimetic α-ketoamides is a broad-spectrum inhibitors of the main proteases of β-CoVs and α-CoVs as well as the 3C proteases of enteroviruses (Zhang et al., 2020c). They made P3-P2 amide bond incorporate into a pyridone ring to enhance the half-life of the compound in plasma and showed good pharmacokinetic results in mice, suggesting that the direct administration of compound to the lungs was possible. Dai et al. designed and synthesized two lead compounds (11a and 11b) targeting Mpro, which bound to Cys145 of Mpro (Dai et al., 2020). These two compounds exhibit a good antiviral effect on SARS-CoV-2 and have no obvious toxicity in SD rats and Beagle dogs, especially 11a.

### Blocking SARS-CoV-2 From Target Cells

#### S Protein

S protein is thought as the most important potential target to stop the SARS-CoV-2 from entering target cells via its neutralizing antibodies or inhibitors. Although the S protein of SARS-CoV-2 and SARS-CoV have an amino-acid se-quence identity of around 77% (Zhou et al., 2020), SARS-CoV-specific neutralizing antibodies (e.g., m396, CR3014) fail to bind with SARS-CoV-2 S protein (Tian et al., 2020). Only the CR3002 can neutralize SARS-CoV-2 when the RBD is in the “up” conformation, the CR3022 can bind to RBD (Yuan et al., 2020). Yuan et al. found that there was a highly conserved cryptic epitope in the RBD of SARS-CoV-2 and SARS-CoV though the analysis of the crystal structure of CR3022 (Yuan et al., 2020). While CR3022 could neutralize SARS-CoV, it did not neutralize SARS-CoV-2 at the highest concentration tested (400 μg/mL). Thus, whether CR3022 can treat COVID-19 remains to be determined.

EK1 is a pan-coronavirus fusion inhibitor that target HR1 domain. EK1C4, a lipopeptide derived from EK1, could protect mice from HCoV-OC43 infection (Xia et al., 2020a), suggesting that EK1C4 could be used for prevention and treatment of SARS-CoV-2 infection. However, at present, a number of *in vivo* and *in vitro* experiments are still needed to assess its safety and effect.

Currently, the precise structure of S protein has been already available (Wang Q. et al., 2020; Wrapp et al., 2020; Yan et al., 2020; Yuan et al., 2020) and Yuan et al. provides molecular insights into antibody recognition of SARS-CoV-2 (Yuan et al., 2020). The new specific neutralizing antibodies or inhibitors of SARS-CoV-2 may be developed based on these insights. However, there is a long way to go before clinical application.

#### ACE2

ACE2 is the receptor of SARS-CoV-2. Theoretically, blocking ACE2 can block the binding of SARS-CoV-2 to cells though ACE inhibitor (ACEI) and angiotensin II receptor blocker (ARB). However, given that the importance of maintaining the homeostasis of blood pressure and the balance of fluid and salts of the RAS (Patel et al., 2017), the safety and effect of using these drugs are still unclear. Besides, using ACEI and ARB can increase ACE2 expression in rats (Gheblawi et al., 2020), suggesting that these drugs may increase the risk of SARS-CoV-2 infection. On the other hand, the activation of ACE2 has a protective role in pulmonary injury (Hernández Prada et al., 2008; Shenoy et al., 2013). Recombinant soluble ACE2 (hrsACE2) not only neutralize the virus but also rescue cellular ACE2 activity (Monteil et al., 2020), further protecting pulmonary injury. The latest evidence showed that clinical grade hrsACE2 reduced SARS-CoV-2 recovery from Vero cells by a factor of 1,000–5,000, demonstrating that hrsACE2 can significantly block early stages of SARS-CoV-2 infections (Monteil et al., 2020). Therefore, ACE2 may be a potential target to treat COVID-19. Before using these drugs, the safety and effect must be assessed carefully and the actual situation of the patients should be fully considered.

#### TMPRSS2

The entry of SARS-CoV-2 into cell entry depends on ACE2 and TMPRSS2 (Hoffmann et al., 2020), and TMPRSS2 is essential for virus spread (Iwata-Yoshikawa et al., 2019). A TMPRSS2 inhibitor approved for clinical use is able to block entry (Hoffmann et al., 2020). Therefore, the TMPRSS2 inhibitor might be a treatment option. Actually, this potential target can block the first step of SARS-CoV-2 infection.

### Targets Against “Cytokine Storm”

#### IL-6

In COVID-19 patients, serum IL-6 is increased significantly and correlates with respiratory failure, ARDS, and poor clinical outcomes (Chen G. et al., 2020; Ruan et al., 2020). Tocilizumab is the IL-6 antagonists that is approved by the U.S. Food and Drug Administration (FDA) for the treatment of CAR T cell–induced cytokine release syndrome. Reportedly, preliminary results from an open-label study of 21 patients with COVID-19 treated with tocilizumab in China are encouraging. Fever subsided in all patients within the first day of receiving tocilizumab. Oxygen requirements were reduced in 75% of the patients (Moore and June, 2020).

#### IL-17

IL-17A, a pro-inflammatory cytokine, can regulate the production of many cytokines, such as IL-6, MCP-1, and G-CSF (Josset et al., 2013). IL-17 is produced by Th17 cells mainly and Th17 cells are increased significantly in COVID-19 patients (Wu and Yang, 2020). Therefore, targeting IL-17 alone or in combination with IL-6 may be an approach to treat COVID-19 against “cytokine storm.” However, the specific relations between IL-6 and IL-17 in COVID-19 patients need to be further studies.

### SARS-CoV-2-Specific Antibodies

SARS-CoV-2-specific antibodies can be detected and used to treat patients. Several studies have reported that some severe and critically ill patients showed clinical improvement by using convalescent plasma contained neutralizing antibodies (Duan et al., 2020; Rajendran et al., 2020; Shen C. et al., 2020; Zhang et al., 2020a). To develop neutralizing antibodies for treating large-scale patients, identifying the SARS-CoV-2-specific antibodies from convalescent plasma of COVID-19 patients is essential. Cao et al. identified 14 potent neutralizing antibodies by high-throughput single-cell RNA and VDJ sequencing of antigen-enriched B cells from 60 COVID-19 convalescent patients (Cao et al., 2020). Among the 14 neutralizing antibodies, BD-368-2 was reported to be the most potent one by the analysis of plaque reduction neutralization test and the *in vivo* experiments of mice. Besides, the Cryo-EM structure of a neutralizing antibody revealed the antibody's epitope overlaps with the ACE2 binding site and the neutralizing antibody can disrupt the ACE2-RBD binding by binding to RBD of S protein competitively (Cao et al., 2020). Wu et al. also identified 4 monoclonal antibodies (B5, B38, H2, and H4) from a convalescent patient (Wu et al., 2020). Among them, B38 and H4 showed complete competition with ACE2 for binding to RBD of S protein and recognized different epitopes on RBD with partial overlap. Besides, the potent SARS-CoV-2-specific antibodies identified by Ju and his colleagues from single B cells of eight SARS-CoV-2 infected individuals could not cross-react with RBD of SARS-CoV and MERS-CoV (Ju et al., 2020). These identified SARS-CoV-2-specific antibodies from convalescent plasma of COVID-19 patients are promising candidates for treatment against COVID-19. However, the data from 173 COVID-19 patients found that a higher titer of antibodies was independently associated with a worse clinical classification (Zhao et al., 2020), suggesting the possible antibody-dependent enhancement (ADE) of SARS- CoV-2 infection. This issue must be paid attention to in the subsequent studies.

Another strategy to treat large number of patients is to collect enough plasma from convalescent donors. In the UK, the Office of Life Sciences, NHS Blood and Transplant (NHSBT) and the Department of Health and Social Care (DHSC) have proposed and planned a new program to collect high volumes of plasma. The work is funded as a new £20 m project by DHSC (Roberts et al., 2020). However, there are many problems and challenges. First, currently, there are only several uncontrolled studies assessing the efficacy and safety of convalescent plasma (Duan et al., 2020; Rajendran et al., 2020; Roberts et al., 2020; Shen C. et al., 2020). Therefore, large-scale trials are needed to assess the efficacy and safety of convalescent plasma. Encouragingly, a recent report of 5,000 patients treated with convalescent plasma demonstrated that convalescent plasma was safe with no obvious cases of antibody-dependent enhancement of disease (Shen C. et al., 2020). Second, new methods are needed to evaluate the quantity and quality of anti-SARS-CoV-2 antibodies obtained from different donors. Third, the optimal doses and time point of convalescent plasma transfusion for different patients has not been determined. Currently, the doses of convalescent plasma transfusion by different studies are different. For example, Duan et al. used 200 mL of convalescent plasma with the neutralizing antibody titers above 1:640 to treat 10 patients (Duan et al., 2020), while Zhang et al. used 2,400 ml of convalescent plasma to treat a 73 years old male patient (Zhang et al., 2020a). Of note, the titers of neutralizing antibodies were variable in different recovered patients (Ni et al., 2020). A recent study found that convalescent plasma treatment could discontinue SARS-CoV-2 shedding but could not reduce mortality in critically end-stage COVID-19 patients (Zeng et al., 2020), suggesting that convalescent plasma treatment should be initiated earlier.

## Conclusion

In the current outbreak of SARS-CoV-2, there is an urgent need for developing the most effective therapy. We reviewed the gene structure, protein function and pathogenic mechanisms of SARS-CoV-2 based on the latest reports systematically, finding that SARS-CoV-2 is easier to spread across species and has stronger ability to spread from person to person compared with other CoVs. Therefore, we proposed some potential therapeutic targets from four aspects based on the gene structure, protein function and pathogenic mechanisms of SARS-CoV-2, including inhibiting important proteases, blocking SARS-CoV-2 from to target cells, important targets against “cytokine storm” and SARS-CoV-2-specific antibodies. However, extensive investigations are still needed to evaluate their safety and effectiveness. In addition, multiple organ function damage is a common feature in severe patients, but the current damage mechanisms are not clear, and thus needed further studies in order to guide clinical management better. In conclusion, many questions regarding the pathogenesis of SARS-CoV-2 are still poor understood and demand further investigation. Especially, the precise mechanism of genetic mutation in SARS-CoV-2 must also be further clarified.

## Author Contributions

HS designed and executed the study. JW and XY collected and analyzed the data, and wrote the manuscript. BW wrote the manuscript. RG and WL made the figures. XX was responsible for language quality. LT reviewed the study. All authors contributed to the article and approved the submitted version.

## Conflict of Interest

The authors declare that the research was conducted in the absence of any commercial or financial relationships that could be construed as a potential conflict of interest.
